# The cascade to pathogenicity in autoantibody-mediated CNS diseases

**DOI:** 10.1093/brain/awag158

**Published:** 2026-05-04

**Authors:** Federico Montini, Elinor Wing, Max Herman, Sean J Pittock, Sebastian Lopez Chiriboga, Eoin P Flanagan, Sarosh R Irani

**Affiliations:** Department of Neurology, Mayo Clinic, Rochester, MN 55905, USA; Center for MS and Autoimmune Neurology, Mayo Clinic, Rochester, MN 55905, USA; Department of Neuroscience, Mayo Clinic, Jacksonville, FL 32224, USA; Department of Neurology, Mayo Clinic, Jacksonville, FL 32224, USA; Department of Neurology, Mayo Clinic, Rochester, MN 55905, USA; Center for MS and Autoimmune Neurology, Mayo Clinic, Rochester, MN 55905, USA; Center for MS and Autoimmune Neurology, Mayo Clinic, Rochester, MN 55905, USA; Department of Neurology, Mayo Clinic, Jacksonville, FL 32224, USA; Department of Neurology, Mayo Clinic, Rochester, MN 55905, USA; Center for MS and Autoimmune Neurology, Mayo Clinic, Rochester, MN 55905, USA; Center for MS and Autoimmune Neurology, Mayo Clinic, Rochester, MN 55905, USA; Department of Neuroscience, Mayo Clinic, Jacksonville, FL 32224, USA; Department of Neurology, Mayo Clinic, Jacksonville, FL 32224, USA; Oxford Autoimmune Neurology Group, Nuffield Department of Clinical Neurosciences, University of Oxford, Oxford OX3 9DU, UK

**Keywords:** autoimmune, encephalitis, neuromyelitis optica, MOGAD, autoantibodies

## Abstract

The discovery of pathogenic neuroglial surface-directed autoantibodies (NGSAbs) has fundamentally transformed clinical neurology, by enabling molecular-level diagnoses in potentially treatable, yet previously unrecognized, diseases. Annual descriptions of novel CNS-targeting antibodies create a continuous stream of new conditions in which to evaluate distinct phenotypes, specific tumour associations and immunotherapy responses.

Alongside this clinical growth, increasing basic knowledge has highlighted origins and mechanisms underlying disease causation, most comprehensively interrogated in the well-established autoantibody-mediated conditions of autoimmune encephalitis (AE), neuromyelitis optica spectrum disorder (NMOSD) and myelin oligodendrocyte glycoprotein antibody-associated disease (MOGAD). The corresponding most common ‘big six’ autoantigens are LGI1, the *N*-methyl-D-aspartate (NMDA) receptor, CASPR2, IgLON5, in forms of AE, AQP4 and MOG. Each of these autoantigens associates with a homogenous set of basic clinical features, across age, sex, tumour associations and ethnicities, coupled with partly distinctive profiles of triggers and predispositions, paradigms of immune tolerance escape in the periphery, how cells and autoantibodies gain access to the CNS, and discrete mechanisms by which the CNS autoantibodies induce neuroglial dysfunction. These observations lead us to reconstruct a proposed chronological series of events as the ‘cascade to pathogenicity’, which together culminate in a rare CNS disease. By extension, we hypothesize elucidating the underlying biology of each condition will present differing precision medicine approaches to optimize patient care. Despite distinctions, there are also clinical and biological overlaps between these diseases, collectively creating opportunities to compare and contrast their individual features.

Here, in each condition, we review current knowledge regarding the similarities and differences between the triggering events, underlying immunological processes and pathogenic mechanisms of autoantibodies. In some instances, we identify scientific clues that drive hypothetical pathways of pathogenesis and, for others, highlight striking observations that aim to generate hypothesis-driven next steps.

Our aim is to construct a model across the major autoantibody-mediated CNS diseases to highlight distinct components of cascades to pathogenicity, which may offer targeted therapeutic approaches to improve patient outcomes, and identify key areas and questions for future research.

## Teaser

Montini *et al.* synthesize evidence across major autoantibody-mediated brain diseases to propose a ‘cascade to pathogenicity’, linking triggers, breakdown of immune tolerance, CNS entry, and antibody-mediated neuroglial dysfunction. Defining shared and disease-specific mechanisms could help guide precision therapies.

## Introduction

Autoimmune neurology is one of the most dynamic and rapidly advancing fields in contemporary medicine. Over the past two decades, the discovery of multiple autoantibodies directed against autoantigens with epitopes expressed on the surface of neurons and glia has transformed our understanding of neuroinflammatory diseases, shifting the paradigm from syndromic descriptions to molecularly precise autoantibody-mediated diagnoses.^1,2^ These neuroglial surface-directed autoantibody (NGSAb)-mediated diseases also carry major pragmatic importance for our patients as they are typically immunotherapy-responsive, and should be considered ‘not to miss’ conditions.^3-5^

**Figure 1:**
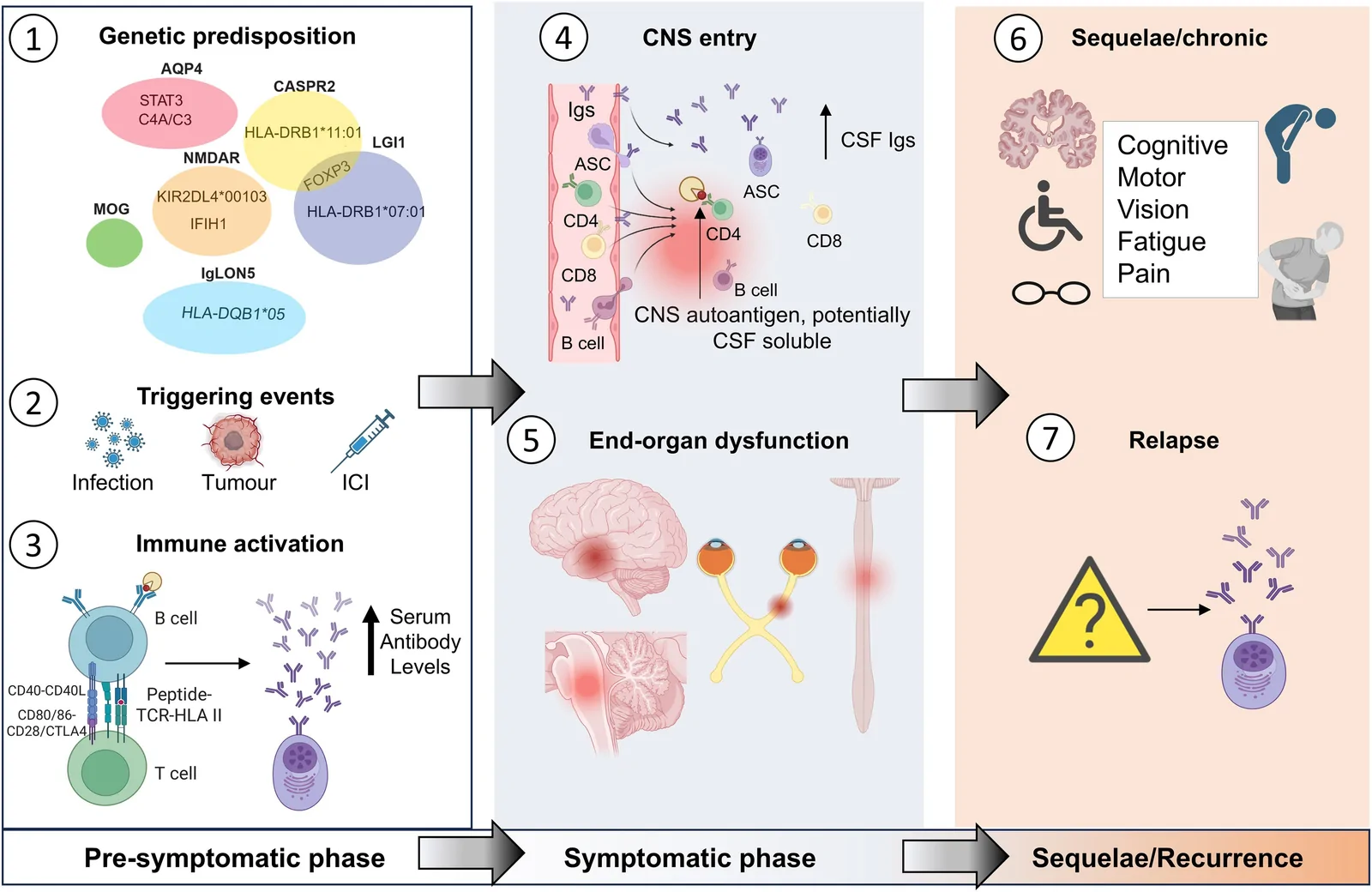
**Cascade to pathogenicity in neuroglial surface autoantibody (NGSAb)-mediated CNS diseases.** Schematic timeline from the pre-symptomatic phase through symptomatic disease to sequelae/recurrence, highlighting a series of events including: (1) genetic predisposition; (2–3) triggering events [e.g. infection, tumour or immune checkpoint inhibitors (ICI)]; (4) CNS entry of autoantigen-reactive lymphocytes/antibodies; (5–7) end-organ dysfunction leading to chronic sequelae and unknown factors precipitating relapse. ASC = antibody-secreting cell; HLA = human leucocyte antigen; TCR = T-cell receptor. Created in BioRender. Montini, F. (2026) https://BioRender.com/k8bgyg5.

NGSAb-mediated diseases encompass a diverse spectrum affecting multiple levels of the nervous system, from the cortex, through subcortical white and grey matter, brainstem, cerebellum, optic nerve, spinal cord and peripheral nerve, resulting in varied clinical phenotypes. While traditional disease classifications often pivot on such anatomical localizations, experiential patterns and clinical courses, the molecular revolution exemplified by NGSAb-mediated diseases offers an opportunity to classify these conditions via their fundamental biological basis.^6^ This molecular-led evolution has already commenced by strictly clinically distinguishing aquaporin-4-antibody-associated neuromyelitis optica spectrum disorder (AQP4 ^+^ NMOSD) from myelin oligodendrocyte glycoprotein antibody disease (MOGAD).^7^ Here, we aim to also distinguish between the varied and clinically divergent forms of autoimmune encephalitis (AE) and incorporate a molecular viewpoint, dissecting triggers and predisposing factors, through key drivers of the aberrant immune system, to mechanisms by which end effector autoantibodies modify neuroglial function and determine patient outcomes (Fig. 1).

Central to a molecular understanding is recognition that these disorders arise from the breakdown of immune tolerance, typically against a single autoantigen. Hence, we propose that B and T cells that recognize this single autoantigen are both sufficient and necessary for pathogenesis of NGSAb-mediated disorders, placing the role of the pivotal autoantigen ‘centre-stage’.^2,8,9^ Their ultimate generation of NGS-reactive autoantibodies leads to diseases, occurring at around 0.2 to 3 per million per year. Our working hypothesis is that such rare and non-inherited diseases likely require a series of multiple more common events to culminate in causation: their ‘cascade to pathogenicity’.^10^

In this review, we aim to apply current knowledge of triggers, immunology and autoantibody–autoantigen interactions to propose a cascade to pathogenicity. Characterizing and interrogating this cascade aims to provide biological insights and multiple therapeutic targets to catalyse the development of molecularly informed precision medicine approaches in NGSAb-mediated diseases. By focusing on the commonest ‘big six’ NGSAb targets (Table 1), leucine-rich glioma inactivated 1 (LGI1), the *N*-methyl-D-aspartate receptor (NMDAR), contactin-associated protein like 2 (CASPR2), immunoglobulin-like cell adhesion molecule 5 (IgLON5), AQP4 and MOG,^11,12^ those most commonly encountered in autoimmune neurological practice, we highlight similarities and differences between the cascades in each condition. Additionally, as the field of NGSAb-mediated diseases is relatively nascent, we selectively incorporate other autoimmune conditions to illustrate concepts of likely pertinence to NGSAb-mediated disorders.

**Table 1:** Clinical and biological features observed in each of the big six neuroglial surface antibody-mediated diseases

Autoantigen	Clinical features						Biological features						
	Age, years, median (range)^a^	Sex (M:F)	Tumours	Main ethnicities	HSV association	Relapses	Subclass	BCR mutations	Complement activation	HLA restriction	Antigen internalization	Steric hinderance	ADCC
LGI1	∼60 (30–95)	2:1	<5%	White	–	++	IgG4 > 1	+++	–	++++	++	+++	–
CASPR2		7:1	10%	White	–	++	IgG4 > 1	+++	–	+++	–	+++	–
IgLON5		1:1	<5%	White	–	+/–	IgG4 > 1	N/A	–	++++	++	+++	–
NMDAR	∼20 (1–80)	1:3	∼30%	Afro-Caribbean and Asian	++	+++	IgG1	+/–	–	–	++++	–	–
AQP4	∼40 (10–70)	1:7	<5%	Afro-Caribbean	–	++++	IgG1	+++	++++	–	–	–	++
MOG	∼35 (5–70)	1:1.5	<5%	White	–	+++	IgG1	+/–	++	–	–	–	++

Strength of association estimated from nil (−) through low (+/++) to high (+++/++++). ADCC = antibody dependent cell cytotoxicity; BCR = B cell receptor; F = female; HLA = human leucocyte antigen; HSV = herpes simplex virus; M = male; N/A = no available data; NMDAR = *N*‑methyl‑d‑aspartate receptor.

^a^Simplified from primary data to represent summary statistics.

## The longitudinal cascade to pathogenicity

**Figure 2:**
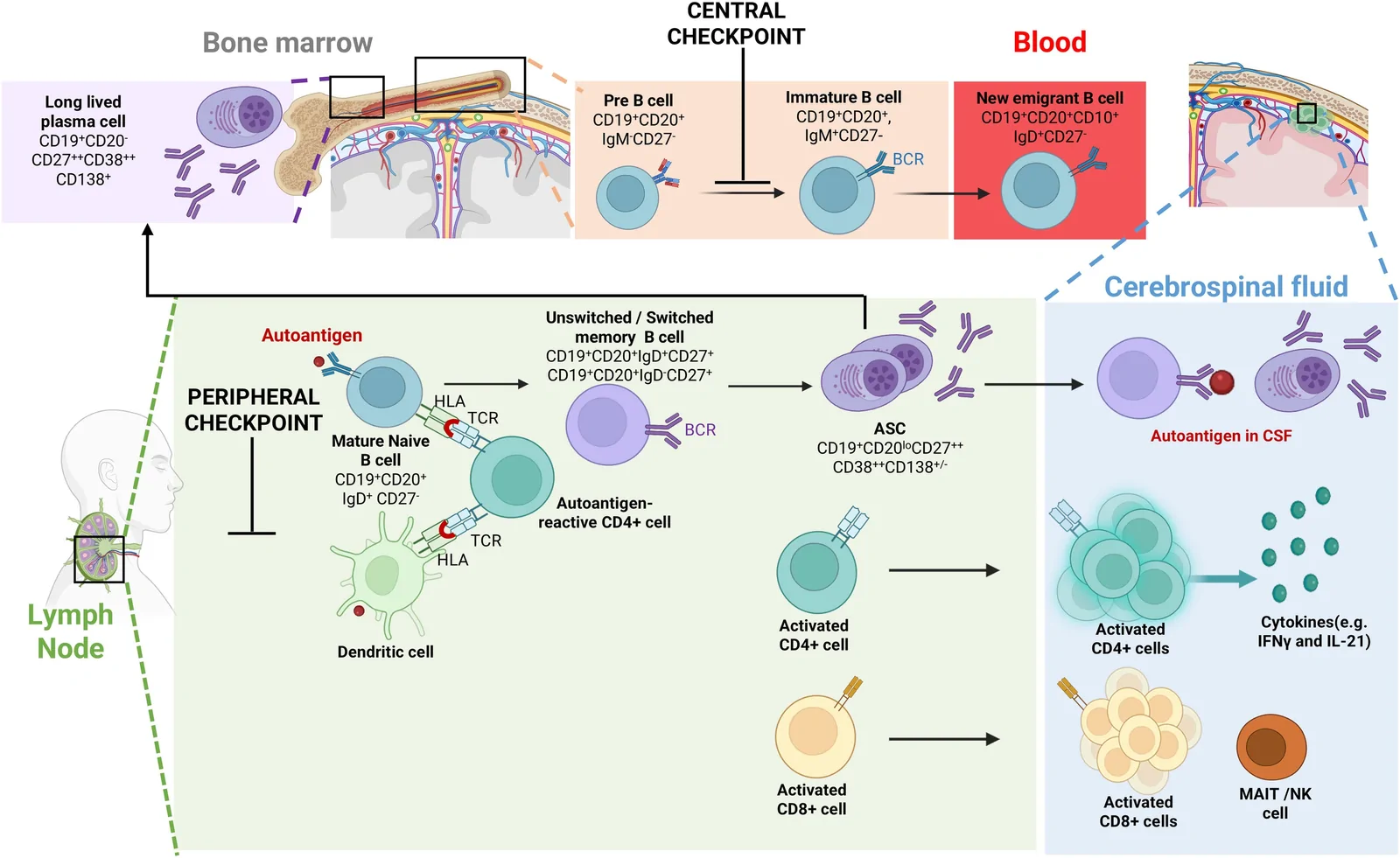
**Anatomical compartments and B-cell checkpoints underlying immune tolerance escape and access to the CSF.** Neuroimmune anatomical compartments, including bone marrow (skull and long bone), blood, lymph node and CSF, and the central and peripheral tolerance checkpoints which shape the B-cell repertoire. In the bone marrow, B-cell development proceeds through preB (surrogate light chain expressed, in red) and immature B cells (CD19^+^ CD20^+^ IgM^+^ CD27^−^, that express a bone fide light chain and hence a mature B-cell receptor, BCR), which flank a central checkpoint. The first B cells to reach blood are new emigrant B cells (CD19^+^ CD20^+^ CD10^+^ IgD ^+^ CD27^−^) and can interact in germinal centres (typically situated in lymph nodes) as mature naive B cells (CD19^+^ CD20^+^ IgD^+^ CD27^−^) after traversing a peripheral checkpoint. Here, their interaction with dendritic cells and autoantigen reactive CD4^+^ T cell signals via the TCR-HLA class II, CD80/86-CD28/CTLA4 and CD40-CD40L molecular pathways alongside cytokines (e.g. IFNγ, IL21). These pathways generate unswitched/switched memory B cells (e.g. CD27^+^ with or without IgD expression, respectively), short-lived plasmablasts/ASCs (CD19^+^ CD20^lo^CD27^++^CD38^++^CD138^+/−^) and long-lived (CD19^+^ CD20^−^ CD27^++^CD38^++^CD138^+^) plasma cells, which secrete autoantibodies. The CSF compartment highlights the infiltration of B cells and ASCs, and contributions from T cells (both CD4^+^ and CD8^+^) and other rare types, including MAIT/NK cells. Soluble antigen (from secretion/ectodomain shedding or injury-related release) may engage BCRs and retain antigen-specific B cells/ASCs in CSF. BCR = B-cell receptor; ASC = antibody-secreting cell; LLPC = long-lived plasma cell; MAIT = mucosa-associated invariant T cell; NK = natural killer; HLA = human leucocyte antigen; IFN-γ = interferon-gamma; IL-21 = interleukin-21.Created in BioRender. Montini, F. (2026) https://BioRender.com/187o6s1.

**Figure 3:**
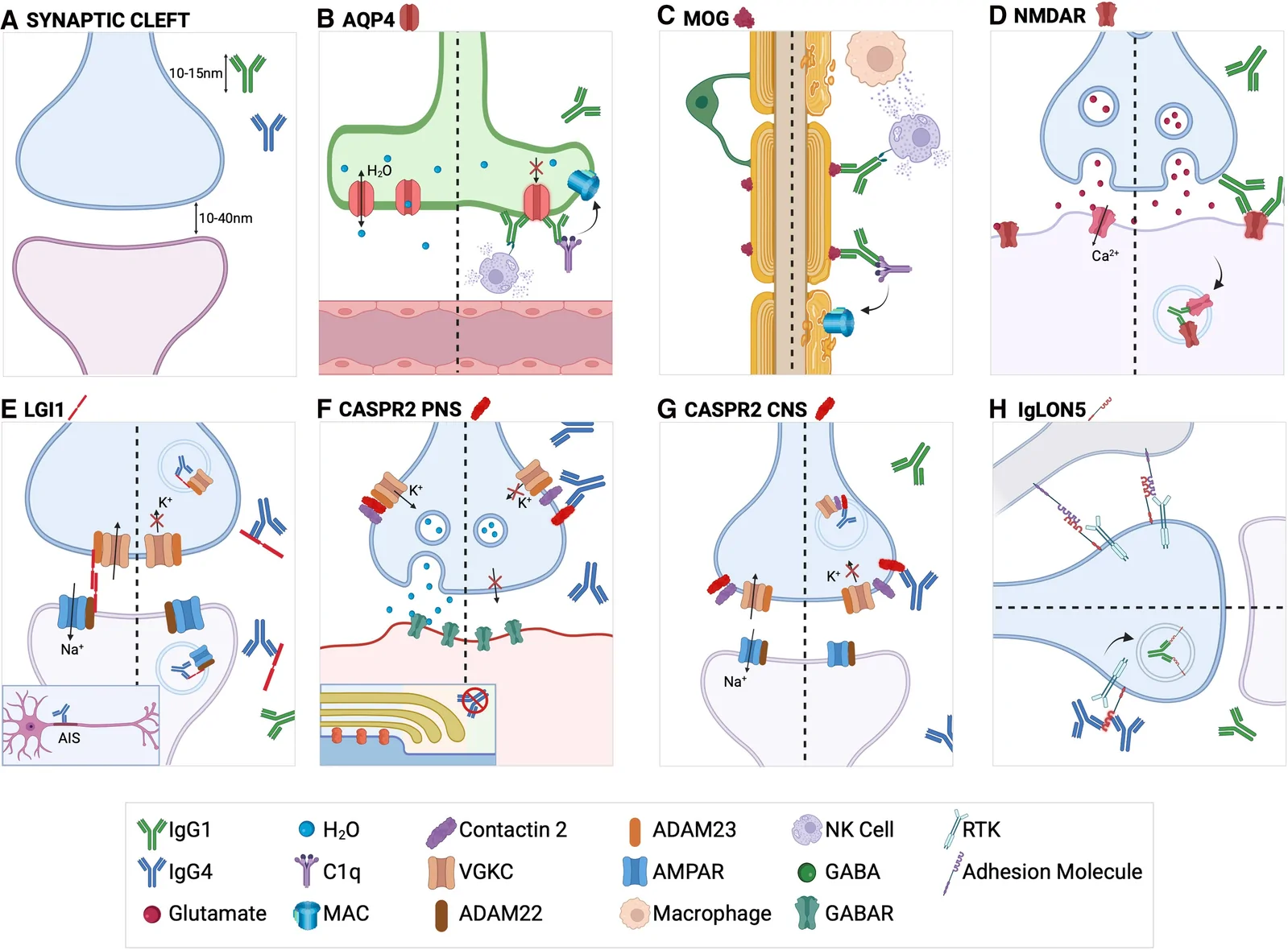
**Mechanisms by which autoantibodies modulate neuroglial surface antigens.** (**A**) Schematic showing the potential discrepancy between size of the synaptic cleft compared with IgG antibodies. (**B**) IgG1 binds AQP4 on the astrocyte end feet, triggering the classical complement pathway and ADCC via NK cells, and other cell types including neutrophils. (**C**) MOG-IgG1 s can function via complement fixation, and promote NK cell-mediated cytotoxicity plus infiltration and activation of macrophages and microglia. (**D**) NMDAR-IgG1 induces receptor internalization leading to NMDAR hypofunction. (**E**) LRR domain-directed IgG4 s can lead to internalization of LGI1 and its neighbouring proteins. Whereas EPTP domain-directed IgG4 blocks the docking of LGI1 with ADAM22/23. Both effects may disrupt pre-synaptic Kv1 potassium channels or post-synaptic AMPARs. Additionally, the axon initial segment is a proven site of LGI1-antibody effects, with potential to modulate action potential frequency (inset). (**F**) IgG access to CASPR2 in PNS juxtaparanodes may be limited by myelin. Pain and other PNS symptoms are more likely caused by effects on unmyelinated sensory nerve terminals. (**G**) CASPR2-IgG4 s may bind synaptic/extrasynaptic CASPR2 leading to internalization of CASPR2, plus disruption of the CASPR2–contactin-2 interaction. (**H**) IgLON5-IgG1 induces internalization of IgLON5, whereas IgLON5-IgG4 likely acts to the block protein–protein interactions antibody; both mechanisms ultimately alter interactions with neighbouring proteins. ADCC = antibody-dependent cellular cytotoxicity; NK = natural killer; NMDAR = *N*-methyl-D-aspartate receptors; LRR = leucine-rich repeat; EPTP = epitempin; ADAM = a disintegrin and metalloproteinase domain-containing protein; AMPAR = AMPA receptor; IgLON = immunoglobulin-like cell adhesion molecule; MAC = membrane attack complex; RTK = receptor tyrosine kinase. Created in BioRender. Montini, F. (2026) https://BioRender.com/7kbps9d.

Consistent with a proposed cascade, we construct a timeline which begins with genetic predispositions, including human leukocyte antigen (HLA) associations and other emerging variants, that likely alter the thresholds or nature of immune tolerance, permitting the escape of NGS-reactive lymphocytes (Fig. 1). Triggering events, such as infectious agents, NGS-autoantigen expressing tumours and immune checkpoint inhibitor therapies, further augment probabilities of breaking immune tolerance and priming of the immune system against these autoantigens. Tolerance breaks, in both central and peripheral B- and T-cell checkpoints, likely leads to the generation of NGS-reactive memory B cells and antibody-secreting cells which gain access to the CNS to secrete pathogenic antibodies, where they have immediate proximity to the extracellular domains of their molecular CNS targets (Fig. 2).^13-15^ Here, the fundamental qualities of the pathogenic antibodies and the nature and site of the target autoantigen interact to manifest varied mechanisms of dysfunction at molecular, circuit and systems levels, ultimately resulting in characteristic clinical phenotypes and disease trajectories (Fig. 3).

## Fundamental phenotypic distinctions closely associate with autoantigen specificities

A central clinical observation is that the autoantigen target forms a pivotal feature to help demarcate fundamental clinical features (Table 1). For example, patients with LGI1-, CASPR2- and IgLON5-antibodies typically present after 50 years of age, and very rarely in childhood.^16,17^ Whereas patients with NMDAR are mostly far younger, often children and young adults, although older onset cases are increasingly well-recognized and often have tumours.^18-20^ Both NMDAR- and AQP4 ^+^ NMOSD show a bias to females and non-Caucasians; whereas MOGAD shows a slight female predilection, rare associations with tumours and no marked ethnic predispositions.^21-23^ Hence, the sex ratios, tumour and infectious associations of these diseases show well-established and geographically reproducible skews which, again, appear to co-segregate with the autoantigen (Table 1). While core symptoms of individual illnesses may be attributed to the dominant CNS localizations of the autoantigen, why should the target autoantigen, the end-product of the cascade to pathogenicity, be positioned to determine such a distinctive range of fundamental demographic and epidemiological associations? This question led us to hypothesize that the corresponding aetiological and underlying biological features of each autoantigen-defined syndrome will show distinctions, a major focus of our review. Yet, particularly given the ongoing and rapid evolution of knowledge in NGSAb diseases, we also identify some overlapping features between these syndromes which suggest parallel pathophysiological mechanisms.

## Predispositions and triggers of NGSAb-mediated conditions: the presymptomatic phase

### Genetic predispositions

#### HLAs

Genetics forms a canonical basis to elucidate fixed risk disease factors. HLA class II associations dominate the known genetic landscape in three of the six core NGSAb-mediated disorders, associated with LGI1, CASPR2 and IgLON5-antibodies (Table 1; Fig. 1). In each of the three diseases, one specific HLA allele is expressed in ∼75%–90% of the patients: HLA-DRB1*07:01 with LGI1 antibodies,^24-26^ HLA-DRB1*11:01 with CASPR2-antibody encephalitis^26,27^ and HLA-DQB1*05:01 in IgLON5-antibody disease.^28^ However, these HLA alleles exist at ∼10%–25% rates in the control population, suggesting multiple other factors must contribute to clinical disease. These three conditions also share two other distinctive and core features: late-onset age and dominance of the rarest immunoglobulin (Ig)G subclass in serum, IgG4, a distinctive triad likely related to fundamental T–B cell interactions. Indeed, HLA class II molecules on professional antigen presenting cells, notably dendritic and B cells, traditionally present peptide antigen fragments to cognate T-cell receptors on autoantigen reactive CD4^+^ T cells.^29^ By providing the key signal mediating this critical interaction, HLA molecules shape the pivotal CD4^+^ T–B cell interaction to initiate or suppress the subsequent immune response (Fig. 1). This interaction likely operates in germinal centres—sites of clustered lymphocytes that act as ‘training grounds’ for T–B cellinteractions, mostly situated in lymph nodes—and can initiate both Ig class switch recombination (CSR), constant gene region exchanges which lead to expression of a different Ig isotype or subclass, and somatic hypermutation (SHM), a process by which targeted mutations are introduced into variable regions of B-cell receptors. These are two of the major mechanisms that focus the immune response against a given antigen.^30^ A single dominant HLA likely suggests T–B cell interactions are markedly biased by few peptides which efficiently bind the specified HLA and that this peptide-HLA complex is then recognized by, likely oligoclonal, T cells. Hence, the dominant HLA associations may reflect limited variation across the CD4^+^-HLA–peptide interactions, presenting a relatively stereotyped drug target for future therapeutic options.

#### Outside of HLA

In LGI1-antibody encephalitis, polygenic risk scores attribute up to 25% of disease risk to genetics.^24^ Yet, *PTPRD* is the only non-HLA gene validated in this condition, with patients very rarely reported to have mutations in *FOXP3*, the canonical transcription factor regulating T-regulatory cell function.^31,32^ NMDAR- and MOG-antibody diseases show weaker or no HLA associations,^33-36^ perhaps suggesting greater polyclonality and promiscuity within the range of potential CD4^+^-HLA-peptide complexes. However, these two conditions also show stronger associations with non-HLA genes (Fig. 1). NMDAR-antibody encephalitis associates with *IFIH1* and specific killer cell immunoglobulin-like receptor (KIR) genes, notably KIR2DL5B*00201.^33,34^ IF1H1 is an intracellular sensor of viral RNA that triggers interferon production, and KIR genes encode proteins expressed principally by natural killer (NK) cells which interact with major histocompatibility complex (MHC) class I molecules. Hence, rather than providing clear T- or B-cell signals to dissect pathogenic pathways, these associations implicate innate immunity in some NGSAb diseases,^9^ supported by rare cases with mutations in IRAK4.^22^ In AQP4^+^ NMOSD, modest associations with HLA-DQ and HLA- DR genes have been identified, with stronger signals from C4A-B complement genes: all encoded on chromosome 6.^36,37^ Interestingly, of the big six NGSAb diseases, the most marked complement deposition is in AQP4^+^ NMOSD tissue, linking genetic-pathological observations with the near complete responsiveness of this condition to complement inhibitors.^38^

In summary, genetic observations offer both confirmatory and hypothesis-modifying observations which highlight fundamental pathways of innate and acquired immune defects as key contributors to NGSAb disease initiation. Yet, familial clustering of neurologic autoimmunity remains rare and individual allele effect sizes are modest, suggesting other major contributions to autoimmune neurology pathogenesis.

### Exogenous triggers: infection, tumours, iatrogenic aetiologies and the microbiome

#### Infection

Among potential environmental triggers, infectious agents can initiate inflammatory responses with subsequent B and T cell proliferation.^39^ Herpes simplex virus encephalitis (HSVE) represents the best-established infectious trigger for AE, occurring in ∼25% of cases, most commonly with NMDAR-antibodies, and particularly in the young.^40-43^ Currently, Japanese encephalitis virus (JEV) is the only other consistently reported infectious trigger for AE.^44,45^ It is intriguing to ask why this response is being initiated by CNS, rather than peripheral, infections.^46^ A plausible model suggests HSVE or JEV induce neuroglial damage which exposes previously CNS-sequestered antigens. Peripheral lymphocytes may never have been tolerized to CNS-restricted autoantigens. Hence these autoantigen-ignorant lymphocytes, likely draining to the periphery via cervical lymph nodes (CLNs), are more prone to escaping tolerance, potentially explaining the necessity for a CNS infection to induce CNS autoimmunity.^47^

The proposed model of peripheral immune priming also aligns with the several-fold higher levels of NMDAR-antibodies typically observed in serum versus CSF,^2,41^ the presence of serum-only NMDAR-antibodies in an additional ∼20% of patients who do not develop symptomatic AE after HSVE, and the higher plasma IFN gene signatures as a predictor of secondary autoimmunity after HSVE.^40^ It remains unclear why the NR1 subunit of the NMDAR is commonly targeted in this process. Explanations may include a high frequency of NR1-reactive naïve B cells, as observed with CASPR2,^48^ or in limited thymic tolerance against the NMDAR, a phenomenon observed for both NR1 and NR2B subunits.^49^ Additionally, it is not understood why other modes of CNS tissue injury, for example stroke, traumatic brain injury or even other brain infections recognized to date,^41,50-53^ do not generate secondary AE. Perhaps this suggests the degree or nature of tissue damage alone is an insufficient trigger, and that a specific inflammatory milieu may be required. This environment may associate with age-related immune alterations to explain the predilection for AE in younger HSVE patients.^40^ The higher rates of serum HSV-IgG in patients with NMDAR-antibody encephalitis without symptomatic CNS infections led to the alternative suggestion, that non-encephalitic HSV exposure may trigger cases of NMDAR-antibody encephalitis.^54^ Yet, the overall high (∼25%–50%) rates of HSV-IgG in healthy controls suggest this exposure itself is insufficient for a rare disease to manifest, but may be a factor in breaking peripheral tolerance, towards a pathogenic cascade. The high HSV-IgG, yet low NMDAR-IgG, seroprevalence suggests that B cell receptor (BCR) molecular mimicry between HSV proteins and NMDARs is also alone insufficient to initiate NMDAR-antibody encephalitis. Explaining this specificity and sequence from infection-through-autoimmunity creates opportunities for research questions to elucidate key pathogenic mechanisms of direct relevance to disease prevention and treatment.

#### Tumours

Autoantigen exposures may also arise in the context of systemic cancer. Indeed, the expression of a shared antigen in both a tumour and in the CNS is a defining feature of a paraneoplastic neurological syndrome (PNS).^55^ Most such paraneoplastic antigens are cytoplasmic or nuclear in localization, suggesting the initiating event is likely to be a T-cell response to surface HLA-expressed antigens or a secondary B-cell response to antigens exposed after necrosis in the local tumour microenvironment.^56,57^ The activation of lymphocytes directed against tumour antigens may be beneficial, supported by evidence that patients with PNS exhibit lower metastatic burden and improved survival compared with cancer patients without PNS.^56,58,59^ Why only a minority of cancer patients develop PNS remains unclear, and provides potential insights into NGSAb disease immune initiation. While tolerance mechanisms typically aim to constrain such autoreactivity, the inflammatory tumour microenvironment and tumour-derived *neo*antigens, to which the immune system has theoretically been ignorant, create ideal scenarios to evade self-tolerance.^60^ For example, the presence of frequent intratumoural genomic alterations in the Yo antigens, CDR2/CDR2L, are described in PNS-associated ovarian carcinomas.^61,62^ Also, in tumours from patients with other PNS (e.g. GABA_B_ receptor- and NMDAR-antibody encephalitis), the tumours show dysplastic neurons,^63^ higher somatic mutation rates and chromosomal gains of antigen-coding genes,^61,64^ all features of autoantigens that may preferentially break immune tolerance. Additionally, in some tumours, the presence of dense immune infiltrates forming tertiary lymphoid structures containing the autoantigen, suggest the organization of cellular architecture may determine the development of PNS.^65-68^ The detection of NMDAR-reactive B cells in these tumours, and soluble NMDAR antibodies in their cystic components, provides a paradigm which suggests the tumour contains all the necessary machinery to generate the end effectors of NGSAb disease.

#### Medications

In addition, a common iatrogenic intervention in oncology—the administration of immune checkpoint inhibitors (ICI)—can also trigger NGSAb diseases, typically within 6–10 weeks of ICI administration.^69^ As the main checkpoint molecular targets, CTLA4 and programmed cell death 1 (PD-1), are principally expressed on regulatory T cells, the mechanism is thought to be via disinhibition of these cells, with consequent B-cell activation.^70^ This process can result in both typical autoantigen-clinical associations and in well-recognized autoimmune neurological syndromes without recognized autoantibodies. Hence, unleashing of endogenous autoreactive lymphocytes with ICIs may be an opportune avenue to discover novel autoantigens and, additionally, to explore novel emergent phenotypes that may not yet associate with autoantibodies.^71,72^ The occurrence of NGSAb-mediated diseases in the post-transplant immunosuppressed setting has also been described, suggesting that other drugs that modify diverse immune functions can also induce these illnesses.^73^

#### Microbiome

Finally, as robustly established in the pathogenesis of multiple sclerosis,^9,74-78^ the gut microbiome is a plausible contributor to the multistep cascade initiating NGSAb diseases. However, limited evidence of this exists to date. Experimental data show that a HLA-DR restricted immunodominant peptide from the AQP4 protein can activate patient T cells, and shows high homology with a *Clostridium perfringens*-derived peptide.^79^ Furthermore, this very organism was over-represented in AQP4^+^ NMOSD patient stool.^80^ This concept suggests the potential for direct molecular mimicry, at the T-cell level. Yet, faecal *C. perfringens* has a high prevalence, and hence this could only provide one step in the cascade to pathogenicity for AQP4^+^ NMOSD. Additionally, early data suggest reductions in short chain fatty acid producers in both LGI1- and NMDAR-antibody patients,^81,82^ consistent with a pro-inflammatory shift in the intestinal environment which may provide a less tolerogenic milieu for disease initiation.

#### Other considerations

Overall, it is very challenging to analyse or estimate the number of hits or total duration of this presymptomatic phase. Other than studying the conversion from HSVE to AE and longitudinal cases administered ICIs, there are few realistic methods to enrich cohorts for presymptomatic or ‘at risk’ individuals. Therefore, prospective studies in these well-defined two cohorts may be fruitful in understanding features that tip the balance to NGSAb disorders. Does it take days, months or years to manifest a NGSAb disease? In ICI-induced disease, time from medication exposure to clinical features is rapid, suggesting a preformed set of autoantigen-reactive T-B cells are primed by the release of inhibition. A contrasting clue comes from patients with acetylcholine-receptor antibody-positive myasthenia gravis who develop AQP4^+^ NMOSD after a median of 16 years.^83^ Yet, in a case report, AQP4^+^ NMOSD occurred only ∼2 months after a bone marrow transplant, with a clear *de novo* detection of both AQP4-IgG and AQP4-IgM,^84^ the latter consistent with a new immune response as observed after exposure to novel pathogens. Overall, marked potential variability exists in the duration from presymptomatic to symptomatic, and may depend upon the nature and vigour of the immune priming event.

## Peripheral autoantigen-reactive lymphocyte priming

After triggering events, autoreactive B cells must escape tolerance mechanisms to generate antibody secreting cells (ASCs) that produce pathogenic NGSAbs.^2^ The log fold higher levels of autoantibodies seen in serum versus CSF strongly implicate the periphery, rather than CNS, as the initiating site of this response.^2^ Important questions that influence our understanding of pathophysiology and potential therapeutic approaches include: where in peripheral B-cell development these checkpoint breaches occur, how and if T cells are involved, which plasma cells secrete the autoantibodies and the relative roles of anatomical neuroimmune compartments in driving these processes.^2^

### Checkpoints and naive B cells

In the bone marrow, developing B cells progress from preB- to immature B-cell stages by traversing a central tolerance checkpoint (Fig. 2). Here, the ‘Goldilocks’ threshold level of reactivity (which is ‘just right’) between the membrane-inserted BCR and autoantigens determines whether a cell will: (i) modestly recognize its autoantigen and survive its journey through B-cell development; (ii) react excessively and die by apoptosis; or (iii) react too much but subsequently successfully edit its light chain so its BCR acquires reduced autoantigen reactivity, facilitating its survival.^2,85,86^ The relative roles of long bones versus, the recently rejuvenated importance of, skull bone marrow in this process have not been investigated in humans, but recent animal work has suggested that physical communication channels between skull bone marrow and CSF may permit early skull bone marrow resident B cells to be rapidly mobilized to the CNS parenchyma and its borders.^46^

After bone marrow egress into the circulation as new emigrant B cells, peripheral tolerance checkpoints further curate the repertoire (Fig. 2). Here, BCR–autoantigen interactions determine survival as a mature naive B cell via apoptosis or anergy, a process by which an autoantigen-reactive B cell becomes functionally quiescent.^2,8,87^ Hereafter, other peripheral checkpoints, including selection processes in germinal centres, may further modify the frequencies of autoreactive B cells.^30,88,89^

**Figure 4:**
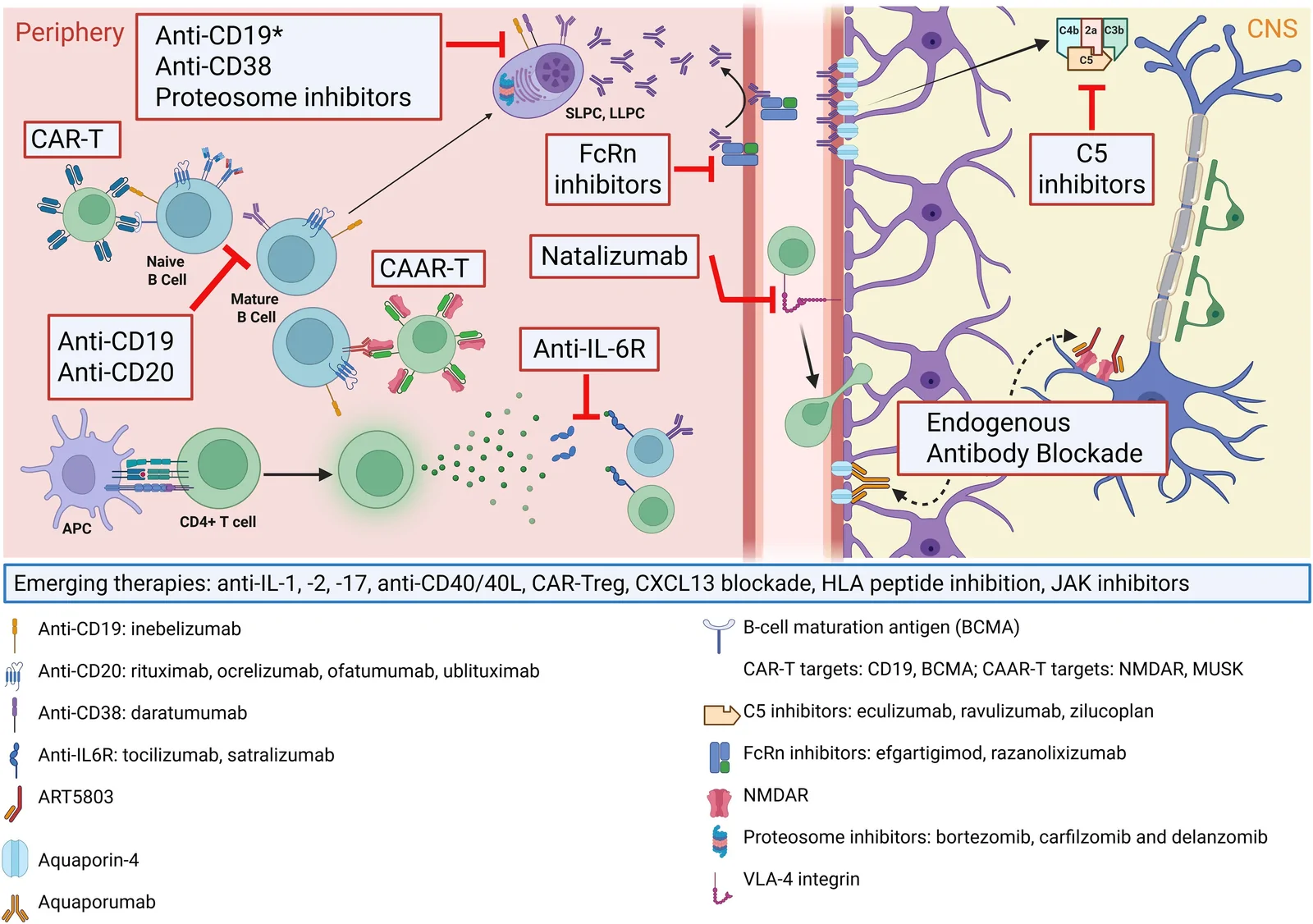
**Therapeutics with targeted mechanisms in autoantibody-driven disease.** Schematic of choice, tailored mechanisms employed in autoantibody neurological disease. Many targeted therapeutics act chiefly in the periphery (*left*), downregulating the immune cells and pathways involved in autoantibody production and maintenance. Most of these (e.g. anti-CD19/20/38, CAR-T/CAAR-T) bind to membrane-bound proteins on pathogenic B-cell or ASC populations to decrease autoantibody production, while others like tocilizumab or FcRn inhibitors target soluble proteins or pathways of antibody recycling to decrease autoantibody production and maintenance, respectively. Natalizumab, acting at the blood–brain barrier (*middle*), is positioned between the peripheral and CNS compartments to prevent B- and T-cell ingress. Medications that act within the CNS at the target site (*right*) serve to prevent autoantibody–antigen interaction and the effector functions therein. Included on the bottom of the figure are emerging therapeutics, highlighting new uses of foundational therapies and emerging medications. *Limited effect on SLPCs, LLPCs. APC = antigen-presenting cell; CAR-T = chimeric antigen receptor T cell; CAAR-T = chimeric autoantibody receptor T cell; FcRn = neonatal fragment crystallizable receptor; HLA = human leucocyte antigen; IL- = interleukin; JAK = Janus kinase; LLPC = long-lived plasma cell; SLPC = short-lived plasma cell; VLA-4 = very late antigen-4. Created in BioRender. Montini, F. (2026) https://BioRender.com/4a73gbm.

The relative integrity of central and peripheral checkpoints have been predominantly examined in primary immunodeficiencies and multiple sclerosis.^8,87^ These studies use generic measurements of autoreactivity, reactivity to Hep-2 cells, double-stranded DNA, insulin and lipopolysaccharide, as their principal readouts. More recently, the presence of reactivity to the specific NGS autoantigens has been determined across these checkpoints. Naive B cells from CASPR2-antibody encephalitis patients and healthy controls both react with CASPR2 at surprisingly high frequencies (∼0.5%), suggesting promiscuous central tolerance in both disease and health. However, CASPR2-reactive memory B cells are identified exclusively in patients, implicating more rigorous later peripheral checkpoints successfully curtail CASPR2 reactivities in health.^48^ By contrast, while AQP4^+^ NMOSD patients harbour naive B cells with AQP4 reactivities, consistent with a breach of the central checkpoint, these are not observed in healthy controls. Hence, tolerance to NGS antigens may be differentially regulated in health.^90^ It is tempting to speculate that B cells in bone marrow rarely encounter the almost exclusively CNS-expressed proteins (such as MOG, CASPR2 and LGI1) but are more likely tolerized against CNS autoantigens which are also peripherally expressed (e.g. AQP4). Future experiments should address this question. However, as no NGSAb-focused studies to date differentiate new emigrant from mature naive B cells, a precise analysis of the conventional checkpoints is currently limited. Nevertheless, unmutated autoantigen-reactive B cells have been isolated in both MOGAD and from NMDAR-antibody encephalitis,^91,92^ and when mutations are removed from patient-derived BCRs against LGI1, CASPR2 and NMDARs, many retain binding against these autoantigens.^15,91^ These collective findings identify naive autoantigen-reactive B cells as a feature common across NGSAb diseases, suggesting a consistent feature of these diseases is the very early loss of tolerance against the key disease-defining autoantigen. This finding also directly implies that deletion of early B-cell populations may represent an under-appreciated, but important, mechanism of action of therapeutics, such as anti-CD19 and anti-CD20 monoclonal antibodies (Fig. 4). Moreover, this explains why haematopoietic stem cell transplantation (HSCT) and B-cell targeting chimeric antigen receptor T-cell (CAR-T) strategies may offer longer-lasting disease resolution (Fig. 4).

### T-cell involvement and germinal centres

In AQP4^+^ NMOSD, and other non-neurological autoantibody-mediated diseases, germline reversion of BCR mutations is reported to abrogate autoantigen reactivity,^93-95^ strongly implying a necessity of SHM for autoantigen recognition, arguing for insufficiency of naive B cells. In line with this, by comparison to mutated LGI1-, CASPR2- and NMDAR-reactive BCRs, loss of mutations consistently decreases affinity for target autoantigens. Hence, despite the importance of naive B cells, SHM likely plays a major role in delivering highly targeted pathogenic autoantibodies. B cells undergo SHM in germinal centres after receiving T cell help via molecular interactions including the peptide-HLA-TCR complex, and costimulatory pathways including CD80/86-CD28/CTLA4 and CD40–CD40LG (Fig. 1). T-dependent pathways represent viable and, in the case of the former, highly specific potential therapeutic targets to terminate germinal centre reactions.^96^ Deletion of the professional antigen presenting cells may be an alternative method to therapeutically terminate peptide-HLA presentation to T cells in germinal centres. Yet, and by comparison to the more pronounced SHM observed in AQP4-, LGI1- and CASPR2-reactive BCRs, the low mutation rates observed in many MOG- and NMDAR-BCRs suggest their receipt of limited T-cell help, and hence restricted participation in germinal centres (Table 1). However, direct T-cell studies to date do not linearly superimpose upon these predictions. While AQP4-, MOG- and NMDAR-reactive CD4^+^ T cells have been directly identified,^79,97-99^ LGI1-reactive T cells were undetectable, creating a vacuum regarding the identity of cells that induce observed mutations in LGI1-reactive BCRs.^99^

Nevertheless, classical germinal centres are typically formed in secondary lymphoid organs, mostly lymph nodes. In mice, those which predominantly drain the CNS are the CLNs,^100^ and receive contents of meningeal lymphatics that carry mixed CNS extracellular fluid and cerebrospinal fluid, and are hence enriched for CNS-derived autoantigens.^101^ Direct sampling of human CLNs has identified high frequencies of NMDAR- and AQP4-reactive B cells, alongside intranodal synthesis of AQP4-IgGs,^102,103^ implicating CLNs as local sites of focused autoantigen-reactive immunizations. Further, human CLNs contain enrichments of multiple CNS-expressed proteins, including neurodegenerative biomarkers such as tau.^104,105^ Taken together, CLNs may provide a hub of neuroimmune autoantigen–lymphocyte communications which initiate and propagate the autoimmunization that culminates in the generation of NGSAbs.

Classical germinal centre activity can generate both memory B cells and ASCs. Additionally, extrafollicular responses, which occur inside secondary lymphoid organs but outside of germinal centres, more rapidly generate short-lived ASCs but these induce relatively transient antibody responses which are typically unmutated, lower affinity and often of the IgM isotype. However, both extrafollicular and germinal centre responses can initiate Ig CSR,^106^ in which IgD and IgM BCRs in naive B cells express one of the downstream immunoglobulin heavy chain genes encoding IgG3, IgG1, IgA1, IgG2, IgG4, IgE or IgA2.^107^ Patterns of immunoglobulin subclasses represent consistent signatures of each autoantigen specificity (Table 1): IgG4-dominant LGI1, CASPR2 and IgLON5 antibodies versus IgG1-dominant NMDAR, MOG and AQP4 antibodies. Subclasses can endow BCRs and soluble autoantibodies with different functional capabilities, such as efficient complement activation with IgM, IgG1 and IgG3, raising the important question of how to mechanistically link autoantigen specificity with the predominant immunoglobulin subclass, discussed further below. CSR can be strategically induced by certain cytokines binding to DNA regions critical to the switching process,^108^ and it may be that NGSAb diseases polarize to certain cytokine patterns.^109^ This may also depend on the immunization microenvironment, for example NMDAR-IgAs predict an underlying ovarian teratoma which typically contains substantial mucosal tissue.^102,110^ While mechanisms of CSR are overall poorly understood they carry future therapeutic importance. For example, the conversion of one subclass to another may be sufficient to nullify pathogenicity of autoantigen-reactive IgGs, as in the case of complement-fixing AQP4-IgG1s.

### Antibody secreting cells

The ASCs generated by germinal centre activity are often classified by their longevity, as either relatively short-lived or long-lived plasma cells (SLPCs and LLPCs, respectively). SLPCs would typically require frequently renewed events of antigen exposure to create sufficient IgG levels to maintain serum autoantibody titres for several years—a phenomenon observed in most NSAb diseases.^111-113^ In this model, either memory B cells would re-enter germinal centres or *de novo* germinal centre activity would be initiated by invasion of naive B cells. In repeat animal immunizations, the latter replacement model appears dominant.^114^ In humans with NGSAb diseases, the frequent presence of NMDAR- and AQP4-IgMs in patient sera may suggest new IgM^+^ naive founder clones are continually invading germinal centres.^102,103^ As AQP4-IgMs are closely correlated with relapse timings, these naive B cells may also have direct clinical pertinence.^103^ Similarly, the balance between a monophasic versus relapsing NGSAb disease course may depend upon the equilibrium established between the generation of SLPCs versus LLPCs. LLPCs usually return to a poorly drug-accessible bone marrow niche where they are non-proliferative and dedicate much energy to secretion of durable, high-affinity immunoglobulins against a single target autoantigen.^2,115,116^ These LLPC-derived antibodies are thought to effectively maintain natural and vaccine-induced immunity against varied organisms, potentially for decades.^2,117^ However, their role in NGSAb diseases, and other autoimmune conditions, has received very little attention, likely as there is limited availability of patient bone marrow for studies. By inference, serum autoantibody levels that do not change substantially after administration of rituximab is consistent with the dominant contribution of CD20^−^CD19^+/−^ LLPCs to serum IgGs. In AQP4^+^ NMOSD, the antibody is considered as lifelong in most patients, implicating its secretion by LLPCs. Clinical quiescence and conversion to seronegative status is observed after haemopoietic stem cell transplantation (HSCT), with virtually no other treatments rendering these patients seronegative.^118^ As chemotherapy prior to HSCT is intended to deplete LLPCs, this observation may strengthen a role for these cells in maintenance of AQP4-IgGs. However, HSCT has wide ranging cellular effects and does not appear to be a permanent solution: exemplified by cases who seroreverted after several years, likely indicating a fundamental predisposition to *de novo* generation of this antibody in AQP4^+^ NMOSD patients.^119^ CAR-T-cell therapies which can target both SLPC and LLPC populations, for example those with CD19 or B-cell maturation antigen (BCMA) specificities, may more robustly reduce autoantibody levels, as suggested by early studies.^120^

## Gaining CNS access

Fundamentally, NGSAbs must reach their target autoantigens in the CNS to be pathogenic. An important question is whether NGSAbs are passively or actively transported into the CNS or whether the peripherally primed B cells are the major traffickers. Also, knowledge around the duration of lymphocyte and antibody retention in the CNS will provide important potential therapeutic implications.

### Cells in CSF

Access mechanisms may vary based on the integrity of the blood–brain barrier. Gadolinium enhancement suggests this is highly permeable in, for example, AQP4^+^ NMOSD where most acute lesions enhance: this creates potential for substances with large molecular weights, such as IgG and IgM to enter the brain parenchyma. Whereas in LGI1-, CASPR2-, IgLON5- and NMDAR-antibody diseases, and 50% of MOGAD cases, no or very little gadolinium enhancement is observed,^121^ suggesting a more stringent and selective mechanism of entry may be necessary. Yet, in all these conditions studied to date, CSF contains a remarkably high frequency of autoantigen-reactive B cells and ASCs, reported at between ∼50%–80% in AQP4-, LGI1- and CASPR2-antibody diseases and ∼10% in NMDAR-antibody disease.^13,15,122^ This suggests a deliberate process is selectively homing these autoantigen-reactive cells to the CSF, where they can then secrete pathogenic antibodies in the close vicinity of their targets. It may be a high local IgG concentration is required for disease. This chemoattraction process may involve soluble factors including cytokines and chemokines, such as CXCL13. CXCL13 is elevated in CSF of patients with NMDAR-antibody encephalitis and its levels correlate with the degree of intrathecal NMDAR-antibody synthesis, a surrogate for the number of CSF NMDAR-reactive plasma cells.^123^ Blocking CXCL13, or other chemoattractants, may represent an attractive therapeutic avenue. Yet, cytokines would not be predicted to specifically retain the autoantigen-specific cells in CSF, but rather retain cells agnostic to their BCR specificity. Hence, the observed enrichments of autoantigen-specific cells in CSF strongly indicates the hitherto unproven presence of soluble autoantigens in CSF. While LGI1, CASPR2, MOG and IgLON5 can be secreted,^124-127^ it is less clear how this process may operate for non-secreted proteins such as AQP4 or the NMDAR.

### Retention of cells in CNS

While attractant signals remain unproven, once in the CSF, B cells may establish tertiary lymphoid structures and a plasma cell niche, as observed in the meninges of patients with multiple sclerosis.^128^ If so, CNS penetrant therapies may be required for treatment of NGSAb diseases. Yet, at least in LGI1- and CASPR2-antibody diseases, most mutations and affinity for the autoantigen appear peripherally acquired, prior to CSF access, suggesting limited autoantigen-reactive B-cell maturation is observed in CSF in NGSAb diseases.^15^ However, given the vast diversity of BCRs, cross-sectional assessments may not detect maturation of individual NGSAb-reactive B cells, implicating longitudinal CSF assessments are required in future studies. Histology may be a gold standard for determining the presence of tertiary lymphoid structures in these diseases, although as mortality is thankfully low, few specimens are available. Yet, to date, histology in MOGAD, AQP4^+^ NMOSD and NMDAR-antibody encephalitis have all reported the absence of meningeal follicles.^129-131^

The role of CSF T cells is less well established and initial studies suggest overall limited clonality in the CSF of LGI1- and CASPR2-antibody patients, suggesting few autoantigen-reactive T cells and aligning with the notion that there is limited help for BCR affinity maturation within the CNS.^15,132^

### Antibody access to CSF

It is also possible that soluble serum IgGs directly access the CSF or CNS parenchyma. In AQP4^+^ NMOSD, lesions can occur around the ventricles, where the blood–brain barrier is incomplete, suggesting that the constitutively expressed antigen may be exposed to pathogenic systemic IgGs.^133^ Yet, for AQP4^+^ NMOSD, a substantial proportion of the CSF immunoglobulin proteome overlaps with CSF BCR sequences, with limited stand-alone contributions from blood, indicating that intrathecal B cells provide much of the soluble CSF IgG.^14^ Nevertheless, the far higher levels of autoantibodies in serum versus CSF provide a suitable gradient for diffusion into CSF, and perhaps this contribution to CSF is greater in scenarios with more disruption of the blood–brain barriers. Yet, even if IgG does access the CNS in high enough concentrations to elicit disease, it is likely that efflux mechanisms will—often rapidly—remove it, an important consideration in the development of CNS-retained monoclonal antibodies.^134^

## Autoantibody-induced end-organ dysfunction

### General principles

Once autoantibodies access the exposed extracellular domains of antigenic targets, they can exert pathogenic effects through several distinct and overlapping mechanisms, each with potential therapeutic implications. Strikingly, the main NGSAb-mediated diseases conveniently divide into those with dominant IgG4 versus IgG1 subclasses of the autoantigen-specific autoantibodies (Table 1), each with fundamentally different biological properties. IgG1 antibodies are bivalent molecules that can both activate the classical complement cascade, culminating in chemoattraction via C3a and C5a and in C5b-9-mediated membrane attack complex (MAC) pore formation with resultant target cell lysis, and antibody-dependent cellular cytotoxicity (ADCC) when the immunoglobulin Fc domains interact with Fc receptors on innate immune cells.^135,136^ In contrast, IgG4 antibodies mediate very limited complement activation or ADCC but possess the peculiar ability to exchange their two halves: a phenomenon termed Fab-arm exchange, which renders many IgG4 molecules functionally monovalent and therefore markedly diversifies their range of potential antigenic targets.^137^ IgG4-dominant NGSAb diseases rarely show only autoantigen-reactive IgG4s, but these are the least prevalent subclass in human serum (∼3% of total IgG), emphasizing the striking skew observed in these conditions. Perhaps given these discrete reported effects, there are no studies that have attempted to quantify these relative effects across multiple NGSAb-mediated diseases. Table 1 aims to estimate the relative effects of complement activation, antigen internalization, steric hinderance and ADCC.

For the big six NGSAb-mediated diseases, intraventricular or intrathecal ‘passive’ transfer of the autoantibodies to experimental animals can reproduce a phenotype and/or histological changes consistent with those observed in the corresponding patients, a traditional confirmation of their pathogenicity.^1,3,138-142^ For AQP4^+^ NMOSD, this requires an additional source of complement, and for MOG the marked differences between rodent and human MOG inevitably limit modelling to the cross-reactive IgGs.^141,142^ While convenient proof-of-concept studies, these simplistic models fail to capture the upstream cellular events, triggers and contributions of factors other than soluble IgG. To partly reproduce such key elements of the human disease, further active immunization models are required to initiate disease at the level of the provoking autoantigen.^143,144^

### AQP4

Despite generalizable characteristics, each of the IgG1-dominant diseases appears to mediate neuroglial dysfunction via different dominant mechanisms. AQP4 antibodies show the capacity to internalize surface AQP4, a property dependent on an intact bivalent IgG molecule facilitating AQP4 cross-linking.^145^ The densely packed arrays of AQP4 on astrocyte end feet facilitate binding of complement (C1q) to multimerized Fc domains.^146^ C1q activation subsequently initiates classical complement pathways resulting in MAC deposition on both astrocyte and neighbouring neuron cell membranes (Fig. 3).^145,147^ Marked complement deposition, alongside abundant IgG and IgM, is observed in AQP4^+^ NMOSD pathology,^148^ tissue which also contains neutrophils, eosinophils and NK cells, providing ideal substrates for ADCC (Fig. 3B). Activated NK cells can release perforin and granzymes, inducing apoptosis, whereas macrophages and neutrophils can phagocytose cells and release cytotoxic mediators such as reactive oxygen species and proteases. Despite all these possible mechanisms, the almost absolute efficacy of complement inhibitors in AQP4^+^ NMOSD suggests this is the major mechanism operating in patients.^149^

### MOG

MOG is expressed on the outer surface of the myelin sheath in the CNS. MOGAD histopathological studies typically demonstrate more limited deposition of complement components than in AQP4^+^ NMOSD, likely because MOG is sparsely expressed on the outer myelin sheath, representing ∼0.05% of the surface proteins, limiting the ability of MOG-IgGs to form the hexameric assemblies required for C1q binding.^150^ Yet, patient-derived MOG-IgGs can strongly induce complement-dependent cytotoxicity (CDC) *in vitro* using cell lines expressing MOG,^136^ perhaps as *in vitro* overexpression produces unnaturally high antigen densities which enable hexamer formation. MOGAD tissue also shows marked infiltration and activation of macrophages and microglia and MOG-IgGs can promote NK cell-mediated cytotoxicity against MOG-expressing cells, where the extent of ADCC correlated strongly with serum MOG IgG levels (Fig. 3C).^151,152^ Further, MOGAD patient tissue contains active peripherally derived macrophages with early cytoplasmic myelin degradation products, including MOG,^153-156^ further implicating the contribution of innate immune cell-mediated mechanisms to demyelination in MOGAD.

### NMDAR

The other common IgG1-dominant NGSAb disease is NMDAR-antibody encephalitis. In this condition, there is limited observed neuronal loss and pathology rarely shows complement products.^157^ Yet, patient IgGs can deposit complement *in vitro* suggesting that, like MOG-IgGs, the human antigen density and distribution may fail to promote C1q binding. Rather, the dominant mechanism of NMDAR antibodies appears to be NMDAR hypofunction induced by internalization by the IgGs, an effect maintained with bivalent Fc-deficient F(ab’)2 fragments but not with monovalent Fab fragments, indicating necessity of both IgG arms to cross-link NMDARs (Fig. 3D).^158^ This effect can occur very rapidly in cultured neurons with markedly altered post-synaptic currents within just 30 min of incubation with patient-derived NMDAR-reactive monoclonal antibodies.^159^ It is plausible that the combination of rapid direct channel modulation together with subsequent internalization of the NMDAR is sufficient for the complex psychiatric and multifocal neurological dysfunction observed in patients with NMDAR-antibody encephalitis.^19,158,160^

Studies of NMDAR-IgGs raise an intriguing broader point regarding IgG access to the autoantigen (Fig. 3A). Reaching targets in the synaptic cleft may be challenging because the cleft is only 10–40 nm wide, and additionally filled with cell-adhesion molecules and extracellular matrix proteins that physically bridge pre- and post-synaptic membranes. Yet IgG is ∼15 nm in size (Fig. 3A). Indeed, a recent study demonstrated that, after 30 min, NMDAR autoantibodies target and internalize extrasynaptic NMDARs, inducing a major reorganization of extrasynaptic membrane proteins which was sufficient to, within hours, destabilize the synapse itself.^161^ This concept of a ‘secondary synaptopathy’ may also apply to the other NGSAb diseases.^161,162^

At a more macroscopic level, antigen distribution likely dictates the observed clinical features. For example, AQP4 density is reported as highest in the optic nerve, spinal cord and area postrema,^163^ the three major sites of symptomatic pathology in AQP4^+^ NMOSD. Less intuitively, this disease manifests with few other CNS sites of pathology and no apparent peripheral involvement, despite widespread AQP4 expression in the CNS, stomach and kidney. It is plausible that these unaffected sites show lower antigen density which prevents sufficient complement fixation, or that endogenous complement regulatory proteins are expressed in these sites.^164^ For other autoantigens, such as LGI1 or NMDAR,^165^ their density is highest in the hippocampus, and many, but not all clinical features of these diseases can be ascribed to hippocampal dysfunction, suggesting wider network involvement is responsible for many of the observed clinical features.^166^

### LGI1

For the IgG4-dominant diseases, complement and ADCC are less likely effector mechanisms. More often, these autoantibodies typically modulate protein–protein interactions. Internalization may be an additional effect of these autoantibodies but perhaps limited if they commonly become functionally monovalent after Fab-arm exchange.^137^ LGI1 is a secreted protein, composed of an N-terminal leucine-rich repeats (LRR) domain and a C-terminal epitempin (EPTP) domain, which forms a dimer or trimer within a trans-synaptic complex, linking post-synaptic ADAM22 to pre-synaptic ADAM23 (Fig. 3E).^167^ Patient-derived monoclonal antibodies have been used to dissect the molecular pathogenesis of this condition: antibodies which bind the LRR domain lead to internalization of LGI1 and its neighbouring proteins, like pre-synaptic K_v_1.1 and post-synaptic AMPA receptors (AMPARs), whereas, EPTP binders block the docking of LGI1 with ADAM22/23, disrupting downstream signalling without internalization (Fig. 3E).^125^ Similar molecular effects have been observed after transfer of patient serum IgGs to the ventricular system of mice, alongside a phenotype consistent with memory loss.^139^ Further, when LGI1-reactive antibodies are intraventricularly infused, rats appear to develop clinical and electrographic seizures alongside altered Kv1.1 expression in the hippocampus.^168^ Non-synaptic mechanisms may also play a role in LGI1-antibody pathogenesis, with proven effects at the axon initial segment conferring the ability to disrupt actional potential initiation and integration of synaptic signals.^169,170^ This complex biology of LGI1, and its multiple targeted domains, make it challenging to propose a simple method to therapeutically counter these pathogenic antibodies. Further, it may be that the minority proportion of LGI1-IgG1 antibodies are more pathogenic. This is suggested by their correlation with cognitive performance and hippocampal sclerosis, and the presence of complement deposition in a few studied post-mortem brain tissues.^171-173^

### CASPR2

CASPR2 is a membrane protein with a large extracellular domain and strongly expressed at the juxtaparanodes of myelinated neurons, in both the CNS and PNS. It is the only one of the big six with a prominent PNS phenotype. Functionally, CASPR2 assists Kv1 channels to correctly localize and mediate repolarization. However, despite dense expression of CASPR2 at juxtaparanodes, shielding of this region by myelin appears to prevent access of antibodies (Fig. 3F). Indeed, a passive transfer model has identified CASPR2 antibodies do not pass the paranodal barrier.^174^ Peripheral unmyelinated sensory nerve terminals may be a more likely target of CASPR2 antibodies, particularly as pain is a common feature in these patients, a strong predictor of long-term disability and these patients often show loss of epidermal small nerve fibres (Fig. 3F).^16,111,175-177^ CASPR2 antibodies can also cause a pain syndrome when transferred to mice, likely through dysfunction of the dorsal root ganglia via Kv1 channels.^178^ These subcellular localizations cannot account for the seizures, cognitive disturbances and movement disorders observed in most patients with CASPR2-antibody encephalitis. Here, it is thought CASPR2 expression at synapses is the pathological target (Fig. 3G). The main proposed mechanisms include internalization of CASPR2, disruption of the proposed interaction between contactin-2 and CASPR2, and the effect of CASPR2 antibodies on AMPARs and post-synaptic hyperexcitability, observed within just a few hours *in vitro* with dependence on the subclass of the CASPR2 monoclonal antibody.^48,138^ Finally, as with LGI1, a role for complement has been raised from human histological findings and may be consistent with the dominance of CASPR2-IgG1s in patients with forms of CASPR2-antibody disease and encephalitis.^179,180^

### IgLON5

Like CASPR2, IgLON5 is a putative neuronal cell adhesion protein with a large extracellular portion and 45%–93% of IgLON5 antibodies are of the IgG4 subclass.^181^ The IgG1 fraction of IgLON5 antibodies reduces surface IgLON5 clusters and, with prolonged exposure, neurofilament disorganization can be induced, an effect not observed with the IgG4 antibodies. It may be that the IgLON5-IgG4 antibodies modify or block neighbouring protein–protein interactions, a more conventional mechanism for IgG4 antibodies (Fig. 3H).^126^ Indeed, the marked early deposition of IgG4s in brain tissue from patients, before overt tau deposition, is consistent with its key role in initiating neuronal pathogenesis.^182^ Later in the disease course, IgLON5-antibody disease patient post-mortem brains typically show tau deposition,^17^ and tau accumulation is reproduced in cultured human and rodent neurons exposed to patient IgG and in passive transfer mouse models, suggesting these antibodies are sufficient to induce features typical of neurodegeneration. This paradigm hence provides a rare and compelling example of molecularly precise immune-to-degeneration directionality in neurology.^140,183^

## How biology shapes resolution, relapse and chronicity

The natural disease trajectory is an additional fundamental feature which is likely best modelled by understanding propagation of the immune processes. Although all these conditions can relapse, tendencies to relapse are likely different, despite being potentially confounded by immunotherapies (Table 1). At one extreme, AQP4^+^ NMOSD is considered a lifelong syndrome, given high recurrence risks after rituximab withdrawal.^119,120,184^ The return of memory B cells and the presence of AQP4-IgM and AQP4-IgG subclass shifts, represent promising indicators of imminent relapses in AQP4^+^ NMOSD,^103,185^ consistent with the concept that clinical attacks arise secondary to recurrent, dynamic germinal centre reactions with continued autoantigen availability and incomplete restoration of tolerance.^2^ Perhaps these reactions reflect T-cell activation licensing escape of AQP4-reactive B cells, and hence both AQP4-reactive T and B cells need to interact for relapses to occur.^97^ By contrast, it is rare for post-HSVE to relapse, even by comparison to NMDAR-antibody encephalitis. Perhaps in this scenario the immune milieu created by acute infection and the infectious trigger itself have been eliminated. Similarly, relapse rates in NMDAR-antibody encephalitis are markedly reduced by teratoma removal,^19,158^ suggesting elimination of a pivotal germinal centre and the associated aberrantly expressed autoantigens are key to remaining monophasic. Alternatively, in this and other long-lived diseases such as NMDAR-antibody encephalitis and some forms of MOGAD, autonomous actions of long-lived plasma cells could be key to persistent autoantibodies and disease activity. However, it is a consistent observation across NGSAb diseases that the NGSAb levels per se show minimal correlations with clinical outcomes, suggesting this population of cells are not driving disease activity.^4,111,112,186^ It is also unclear whether recurrent CSF entry is required for relapses or ongoing disease activity, or if immune cells take up more permanent residency in the CNS, a concept which feeds into the tertiary lymphoid structure discussions above.

While the autoantigenic cascade forms the core of the pathology in most NGSAb diseases, additional complexity may exist beyond these observations. The pathogenic pathways may begin as autoantigen specific but can evolve to affect large numbers of neurons or glia, leading to a cascade of more generic events associated with cell damage and network dysfunction. Examples include the diffuse involvement of white matter tracts and regions outside of the hippocampus in LGI1-antibody encephalitis,^166,187^ the move from an astrocyte-focused injury to neuronal damage in AQP4^+^ NMOSD^145,147^ and the development of clinical features that may be more loosely related NMDAR dysfunction, including the movement disorders, in NMDAR-antibody encephalitis. These downstream molecular cascades have received limited study to date but may represent important methods to assess mechanisms of longer-term injury which markedly impair quality of life across these diseases.^111,188,189^

## Therapeutic targets along the cascade

### Current practice

As mentioned above, these fundamental immunological and neurobiological observations can provide targeted avenues for precision therapeutics (Fig. 4). This is of key importance as clinical trials have only yielded US Food and Drug Administration (FDA)-approved therapies for one of the NGSAb-mediated disorders, AQP4^+^ NMOSD, and several clinical trials ongoing in the other diseases have already failed to recruit adequately.^190^

Historically, the treatment of autoimmune neurological diseases has relied upon drugs with broad mechanisms of action, across lymphoid and myeloid lineages and often, both neurons and glia. These medications often target the generic machinery of cell replication (e.g. azathioprine, mycophenolate mofetil) or downstream signalling pathways (e.g. corticosteroids) common to multiple cell lineages, resulting in both broad immunosuppression, CNS activity and significant multi-organ adverse effects.^191^ In the acute phase of treatment, the myriad actions of corticosteroids, intravenous immunoglobulins and plasma exchange may be advantageous in inhibiting multiple pathways, particularly if the precise diagnosis is not yet known. Second-line acute or maintenance therapies, often in cases of more severe and refractory presentations, include other broadly acting agents including deletion of the whole B-cell lineage with CD19 or CD20 targeting medications (e.g. inebilizumab or rituximab, respectively), blockade of the highly pleotropic IL-6 receptor (tocilizumab and satralizumab) or the anti-proliferative cyclophosphamide.^191,192^ The best example of a more targeted therapeutic used in routine clinical practice are the C5 complement protein inhibitors, which almost entirely eliminate relapses in patients with AQP4^+^ NMOSD.^38,149^

However, many of these drugs remain broadly immunosuppressive or, if more targeted, are associated with specific and/or serious side effects.^190,193^ In an attempt to reduce these adverse effects, the underlying biology has illuminated current and future options with likely greater precision which work by targeting: (i) pathways involved in autoantibody production and maintenance; and (ii) autoantibody effector functions (Fig. 4).

### Targeting pathways of autoantibody production and maintenance

Many utilized and proposed medications for NGSAb diseases target the pathways of B-cell differentiation and function as this cell line is most directly implicated in autoantibody production and therefore, disease pathogenicity. Anti-CD20 monoclonal antibodies (e.g. rituximab, ocrelizumab, ublitixuimab, ofatumumab) target B cells in their life cycle from pre-B cells to memory cells and some ASCs which retain CD20, therefore not deleting plasma cells or some of the earliest bone marrow resident B cells (Fig. 2).^194^ The extent to which these agents can mitigate disease activity may depend on several immunobiological factors, particularly which aberrant B-cell population is implicated in driving the ongoing generation of NGSAb, the immunoglobulin subclass and the disease process. For example, the success of rituximab in AQP4^+^ NMOSD may relate to its ability to abrogate germinal centre B-cell activity,^95^ while in LGI1-antibody encephalitis, its more limited reported efficacy may be due to its inability to delete most ASCs or penetrate CSF to target B cells.^12,173^ Intriguingly, there are also some reports of CD20 expression on select T cells, an additional unforeseen mechanism by which these drugs may show clinical efficacy.^195^ Anti-CD19 therapeutics target a broader range of the B-cell lineage than anti-CD20 agents, creating value added in diseases where plasmablasts, SLPCs and LLPCs are implicated in pathogenic autoantibody production.^196^ Similarly, anti-CD38 medications (e.g. daratumumab) and proteosome inhibitors (e.g. bortezomib) can somewhat selectively target these plasma cell populations, making them useful in pathologies where the activity of LLPCs and CSF-resident ASCs may drive ongoing disease.^190,197,198^

Natalizumab targets the alpha4beta1 integrin to block activated lymphocytes from entering the CNS, a process which appears to be important across NGSAb disease pathogenesis given the enrichments of autoantigen-specific B cells observed across multiple NGSAb diseases.^13,15,122^ Additionally, as the alpha4beta1 integrin is expressed by B and T cells, it may be broadly effective against multiple immunological pathways which contribute to pathology.^199^

More recent attention has turned towards CAR-T therapies targeting CD19, CD20 and B-cell maturation antigen (BCMA). This intervention has reported success in individual neurological cases with myasthenia gravis, MOGAD, AQP4^+^ NMOSD and stiff person syndrome, plus a promising phase 1 trial of BCMA CAR-T in AQP4^+^ NMOSD and an ongoing clinical trial of CD19 CAR-T for refractory stiff person syndrome (NCT06588491).^200^ By comparison to therapeutic antibodies, CAR-T cells targeting the same proteins may offer greater penetration into immunologically privileged spaces, such as the CSF and secondary lymphoid organs, resulting in a deeper, more complete depletion of the B-cell lineage. Indeed, frequent occurrence of CAR-T-induced immune effector cell-associated neurotoxicity syndrome (ICANS) is considered a significant adverse effect, although may be less frequent outside of haematological cancer settings as substantially fewer target cells are depleted. Nevertheless, as ICANS associates with CAR-T penetration into the CSF it suggests a mechanism by which CAR-T cells may, somewhat paradoxically, provide enhanced efficacy in CNS diseases.^201^ Despite this enthusiasm, side effects and off-target effects of CAR-T can be severe, including neurological sequelae of encephalopathy, seizures, aphasia and parkinsonism.^202^

Therapeutic monoclonal antibodies against cytokines have been used throughout multiple medical disciplines. Interleukin-6 is a highly pleotropic cytokine with effects including modification of blood–brain permeability, neuronal and glial functions and T cells and plasmablasts.^203^ Blockade of the IL-6 pathway reduced relapses in a randomized trial of AQP4^+^ NMOSD, and has shown promise in refractory MOGAD and AE.^203,204^ Hence, IL-6-IL-6R pathway blockade provides an example of a broad mechanism of action which confers efficacy across several diseases. Identifying cytokines more specific to NGSAb diseases may provide more targeted future opportunities.^109^

Rather than target autoantibody production mechanisms, Fc receptor neonatal (FcRn) blockers, such as efgartigimod and rozanolixizumab, aim to treat disease by blocking antibody uptake into monocytes and/or endothelial cells and hence, increase antibody degradation in circulation.^205^ Clinically, they have proven efficacy in refractory myasthenia gravis and in an exploratory cohort of NMDAR-antibody encephalitis, and are the subject of a Phase 3 clinical trial in MOGAD (NCT05063162).^206^ These drugs reduce autoantibodies proportional to total IgG levels, typically inducing ∼70% reductions in serum IgG levels. Hence, while they should be effective across all autoantibody-mediated conditions, they also lack a truly precision approach to exclusively targeting the *auto*antibodies. Additionally, reports of infections when using FcRn inhibitors, such as enterovirus meningoencephalitis, mandate future vigilance.^205^

One idea to achieve greater precision is targeting of the CD4^+^-HLA–peptide interaction, particularly when the HLA is homogenous within a disease population (Table 1). A consistent HLA-peptide complex may provide exquisite selectivity without impairing the other physiological functions of that HLA molecule, and may effectively terminate the T-cell help required for germinal centre activity.^207^ This approach should be considered within future T-cell studies in NGSAb diseases. Similarly, through limiting T–B cell interactions, blockade of the CD40-CD40L pathway may turn off germinal centre reactions, and has already shown efficacy in rheumatological diseases.^208^

Another precision concept has been to express the autoantigen on T cells, building on the CAR-T approach, this cellular therapy is termed chimeric autoantibody receptor (CAAR)-T cells. The autoantigen expressed on the surface of a CAAR-T cell should selectively interact with and delete autoantigen-reactive B cells. Preclinical models have been promising in desmoglein-3-associated pemphigus, muscle-specific kinase myasthenia and in NMDAR-antibody encephalitis.^209-211^ However, it is plausible that autoantigen-expressing cells will be overwhelmed by soluble serum autoantibodies, rendering the CAAR-Ts inert. Also, the administration of exogenous human autoantigens may inadvertently re-invigorate the immune response, leading to re-immunization of the patient against the autoantigen. Clinical studies are awaited to evaluate these theoretical benefits and risks of this theoretically elegant approach.

### Targeting pathogenic effector functions of autoantibodies

To date, some of the most refined precision therapy opportunities stem from targeting the autoantibody–autoantigen interactions and the autoantibody effector functions as these represent a highly discrete process in NGSAb diseases (Fig. 4). An exemplary case of this concept comes from the development of complement inhibitors in NMOSD, which leveraged the importance of the complement pathway observed in preclinical studies^142,212^ to pave the way for the success of the C5 inhibitors eculizumab and ravulizumab.^38,149^ As with most therapeutic monoclonals, only a tiny percentage can be detected in CSF and this concentration appears sufficient to mediate complement inhibition.^213^

Analogous to a more precise form of FcRn inhibition, selective degradation of the autoantigen-reactive antibodies may be achieved by creating autoantigen-Fc fusion proteins which spare all IgGs, focusing on depleting the autoantigen-specific IgGs.^214^ This concept, as recently demonstrated as feasible for NMDAR antibodies,^215^ would theoretically represent therapeutic opportunities with very few adverse effects which could be applied across all NGSAb diseases.

The autoantibody-autoantigen complex itself may also be a therapeutic target. Aquaporumab is a non-complement fixing monoclonal antibody with sufficiently high affinity for the extracellular domain of AQP4 that it prevents binding of endogenous patient AQP4-IgGs and, by not binding C1q, protects from complement-dependent cytotoxicity.^216^ A similar, yet alternative approach has been taken for the NMDAR using a monovalent engineered antibody which binds with high affinity to the NR1 subunit of the NMDAR, the known immunodominant region of patient IgGs, but without internalizing the receptor or inducing electrophysiological effects.^217^ Importantly, its peripheral administration to marmosets appears to reverse patient IgG-induced behavioural deficits. Again, while clinical efficacy is awaited, this concept is of great interest. Yet, it remains to be seen whether it shows sufficient CNS penetration and can out-compete pre-existing antibodies formed secondary to an ongoing vigorous downstream cellular immune response. Nevertheless, it may prove valuable in preventing the action of preformed autoantibodies while simultaneously tackling the cellular response with corticosteroids, rituximab and other more traditional approaches. In a similar vein, the use of an allosteric activator of the NMDAR has been shown to have efficacy in a preclinical model of established NMDAR-antibody encephalitis, and can antagonize the effects of patient IgGs on NMDAR membrane organization.^143^ Hence, emerging options to exploit the inherently fastidious interaction between autoantibody and autoantigen are exciting novel approaches to identify precision therapeutics in NGSAb diseases.

Finally, there is increasing interest in exploring how to re-deploy innate surveillance mechanisms to restore tolerance. In this vein, peptide-loaded tolerogenic dendritic cells are an additional autoantigen-specific approach which has been proposed in multiple sclerosis and AQP4^+^ NMOSD,^218^ but could be of interest in other NGSAb diseases with probable ongoing peripheral immune drivers.

## Missing pieces and future directions

The detection of NGSAbs confers a molecularly precise diagnosis, multiple coherent fundamental clinical observations and potential for clinical reversibility, elevating the importance of these conditions. Their discovery has not only revolutionized neurological diagnosis in ‘not to miss’ conditions, but also enabled the development of FDA-approved therapies in AQP4^+^ NMOSD. In addition, it is likely they will have wider implications for other associated illnesses, for example cancers, as the anti-tumour paraneoplastic response may be protective.^55,219^ However, the recruitment of patients with rare diseases and the interpretation of clinical trials with differing prior immunotherapies and stages of disease will likely prove a major challenge to interpret or apply in real-world practice. Hence, a major opportunity presented by these conditions is the development of precision therapies, based on the autoantigenic target. Such autoantigen-specific approaches are numerous and would ideally aim to restore immune tolerance, fundamentally creating an exclusive reset of the autoantigen-dedicated immune system and avoiding less specific therapies. The optimal method to reset each of these diseases may differ or show parallels, a future aim to resolve as more research is undertaken into studying their relative immunobiologies. We optimistically anticipate a future akin to molecular diagnostics in oncology, where specific immune findings such as HLA associations, rogue B-cell populations and immunodominant epitopes can be defined and exploited in patients for diagnostic, prognostic and therapeutic benefits. These experiences can be applied to the multiple emergent new autoantibodies in neurological and non-neurological diseases, towards the ultimate, and increasingly realistic, aim of cure.
